# Apelin Enhances Directed Cardiac Differentiation of Mouse and Human Embryonic Stem Cells

**DOI:** 10.1371/journal.pone.0038328

**Published:** 2012-06-01

**Authors:** I-Ning E. Wang, Xiang Wang, Xiaohu Ge, Joshua Anderson, Michael Ho, Euan Ashley, Jianwei Liu, Manish J. Butte, Masayuki Yazawa, Ricardo E. Dolmetsch, Thomas Quertermous, Phillip C. Yang

**Affiliations:** 1 Division of Cardiovascular Medicine, Department of Medicine, School of Medicine, Stanford University, Stanford, California, United States of America; 2 Department of Pediatrics, School of Medicine, Stanford University, Stanford, California, United States of America; 3 Department of Neurobiology, School of Medicine, Stanford University, Stanford, California, United States of America; Instituto Butantan, Brazil

## Abstract

Apelin is a peptide ligand for an orphan G-protein coupled receptor (APJ receptor) and serves as a critical gradient for migration of mesodermal cells fated to contribute to the myocardial lineage. The present study was designed to establish a robust cardiac differentiation protocol, specifically, to evaluate the effect of apelin on directed differentiation of mouse and human embryonic stem cells (mESCs and hESCs) into cardiac lineage. Different concentrations of apelin (50, 100, 500 nM) were evaluated to determine its differentiation potential. The optimized dose of apelin was then combined with mesodermal differentiation factors, including BMP-4, activin-A, and bFGF, in a developmentally specific temporal sequence to examine the synergistic effects on cardiac differentiation. Cellular, molecular, and physiologic characteristics of the apelin-induced contractile embryoid bodies (EBs) were analyzed. It was found that 100 nM apelin resulted in highest percentage of contractile EB for mESCs while 500 nM had the highest effects on hESCs. Functionally, the contractile frequency of mESCs-derived EBs (mEBs) responded appropriately to increasing concentration of isoprenaline and diltiazem. Positive phenotype of cardiac specific markers was confirmed in the apelin-treated groups. The protocol, consisting of apelin and mesodermal differentiation factors, induced contractility in significantly higher percentage of hESC-derived EBs (hEBs), up-regulated cardiac-specific genes and cell surface markers, and increased the contractile force. In conclusion, we have demonstrated that the treatment of apelin enhanced cardiac differentiation of mouse and human ESCs and exhibited synergistic effects with mesodermal differentiation factors.

## Introduction

Myocardial infarction, a common presentation of ischemic heart disease, results in an irreversible necrosis of cardiomyocytes [1]–[3]. This usually results from an acute interruption of blood supply, which is most often caused by atherosclerotic plaque rupture with thrombus formation in a coronary vessel [1]. The most common sequela of this disease process is heart failure (HF), which is the leading cause of hospital admission in the United States for both men and women [4], [5]. With approximately 400,000 annual incidences, coronary heart disease is responsible for 1 in 6 deaths in the US in 2008 [2]. Of the 3 million people suffering from advanced HF, 15–25% await heart transplantation. However, heart organ donation is limited to 1,000 recipients per year [6]. Despite advances in medical, percutaneous, and surgical interventions during the past decades, the 5-year survival of patients with advanced HF still remains around 50%. Therefore, an alternative therapeutic strategy aimed at sustained and permanent myocardial restoration is necessary.

Heart failure, resulting from myocardial injury and subsequent contractile dysfunction, is due to the limited mitotic capacity of terminally differentiated cardiomyocytes [4]. During this disease process, cardiac progenitor cells have been reported to migrate to the injury site, differentiate into cardiomyocytes, and eventually, regenerate the myocardium [7]–[8]. However, the native population of cardiac progenitor cells is extremely limited and decreases significantly during the aging process, compromising its repair potential [9]. In order to replace the lost cardiomyocytes and regenerate the myocardium, cell transplantation is emerging as a potentially useful therapeutic strategy to restore cardiac function. Embryonic stem cells (ESCs) represent a promising source of pluripotent stem cells for cardiac regeneration capable of self-renewal and robust cardiac differentiation [10]. Multiple pre-clinical research efforts have shown the beneficial effects of ESC transplantation therapy in myocardial regeneration [11], [12]; however, its clinical implementation has been hampered by teratoma formation of the undifferentiated ESCs [11]. Although cardiac differentiation protocols have reported high percentage of contractile ESC-derived embryoid bodies (EBs), the percentage of cardiac cells remain low, the effects of the differentiation factors are unclear, and the results are difficult to duplicate [13]–[15]. In a recently reported protocol, induction of hESCs with BMP-4, activin-A and basic fibroblast growth factor (bFGF) generated cardiac, endothelial and vascular smooth muscle cells [16]. The differentiated EBs exhibited contractile cardiac phenotype and cardiac-specific markers.

In this study, we incorporate a novel peptide, apelin, a newly identified peptide ligand for an orphan apelin G-protein coupled receptor (APJ receptor) with 31% sequence similarity to the angiotensin receptor AT1 [17]–[18]. During cardiac development, apelin serves as a critical gradient for migration of mesodermal cells fated to contribute to the myocardial lineage [19], [20]. Zebrafish pregastrulae embryos are shown to express AT1 early in the blastoderm margin and later in regions where cardiac progenitor cells reside. Apelin, a ligand expression, is restricted to this midline, and depletion of the AT1 receptor results in a reduced number of myocardial progenitor cells and absence of an organized cardiac structure. After a myocardial infarction, an up-regulated expression of apelin at the injury site of the left ventricle has been reported, resulting in the recruitment of the cKit+/Flk1+ cardiac progenitor cells and myocardial regeneration [21]. Furthermore, these APJ transcripts are also detected in the developing heart during embryonic growth of mouse and frog, suggesting the involvement of apelin in embryonic myocardial development, regeneration, and function [22]–[23]. This study investigates the effects of apelin in enhancing cardiac differentiation of mouse and human ESCs through selective aggregation of cardiac lineage committed cells.

## Materials and Methods

All reactions were done at room temperature unless otherwise noted.

### Cells

Mouse ESC (mESC) line, TL1, was derived from 129Sv/J mice (kindly given by collaborator Dr. Thomas Quertermous) [24] and maintained on a confluent feeder layer of irradiated mouse embryonic fibroblasts CF-1 line (MEF, GlobalStem, Inc.) in the high glucose Dulbecco's Modified Essential Medium (DMEM, Gibco, Carlsbad, CA) with 15% fetal bovine serum (FBS, Hyclone, Logan, Utah), 1% non-essential amino acids, 1% penicillin/streptomycin, 0.05 mM β-mercaptoethanol (β-ME, Millipore, Billerica, MA) and 1000 U/ml leukaemia inhibitory factor (LIF, Millipore). Human ESC (hESC) line, H7 (commercially available at WiCell, Madison, WI), was maintained with a feeder layer of irradiated MEF CF-1 line at 25% confluency on 0.2% gelatin-coated surfaces. Human ESCs were cultured in the KnockOut DMEM (Gibco) with 20% KnockOut Serum Replacer (Gibco), 1% non-essential amino acids, 1% penicillin/streptomycin, 0.05 mM β-ME, 2 mM L-glutamine (Gibco) and 4 ng/ml basic fibroblast growth factor (bFGF, Invitrogen). Cells derived from 20–60 passages were used for the study. The cultures were grown at 37°C and 5% CO_2_ in a humidified normoxic environment.

### Mouse and Human ESC Cardiac Differentiation Cell Culture Methods

**Figure 1:**
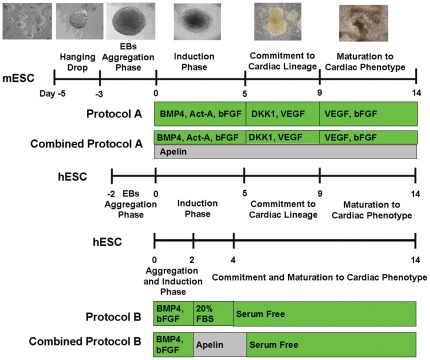
Timeline. Our combined protocols for both mESCs and hESCs include the addition of apelin (grey) to the reported protocols (green). Protocols A and B are indicated in the green boxes without apelin treatment.

To induce differentiation, mESCs were suspended in mouse basal differentiation medium containing 20% FBS, 1% non-essential amino acids, 1% penicillin/streptomycin and 0.1 mM β-ME in DMEM. Then drops (20 µL) of 500 cells were placed on the internal surface of a 100-mm tissue culture dish lids containing phosphate buffer saline (PBS) [25]. After 2 days, the mESCs were aggregated in hanging drops called embryoid bodies (EBs) and were transferred to suspension culture in 96 well ultra low attachment plates for an additional 3 days in mouse basal differentiation medium to enhance the aggregation. 5 day-old mESC-derived EBs (mEBs) were then plated onto 0.1% gelatin-coated 48-well tissue culture plates at a density of one mEB per well. Various differentiation factors were then added into the basal differentiation media at this point (day 0). The timeline for the differentiation is shown in Figure 1.

For hESC differentiation studies, hESC colonies were detached from the Petri dish by using collagenase type IV (Gibco) without dissociating the hESC into single cell suspension. The MEF and hESC colonies were separated by low speed centrifugation (0.5 RPM). Pure population of hESC colonies were allowed to aggregate to form EBs (∼2000 cells/colony) by incubating in 6 well ultra low attachment plates for 2 days in human basal differentiation medium containing 20% FBS, 1% non-essential amino acids, 1% penicillin/streptomycin, 0.05 mM β-ME, 2 mM L-glutamine in KnockOut DMEM. After 2 days, hESC-derived EBs (hEBs) were plated onto 0.2% gelatin-coated 12-well plates at a density of ∼15 hEBs per well. Various differentiation factors were added into the basal differentiation media at day 0 (Figure 1).

**Figure 2:**
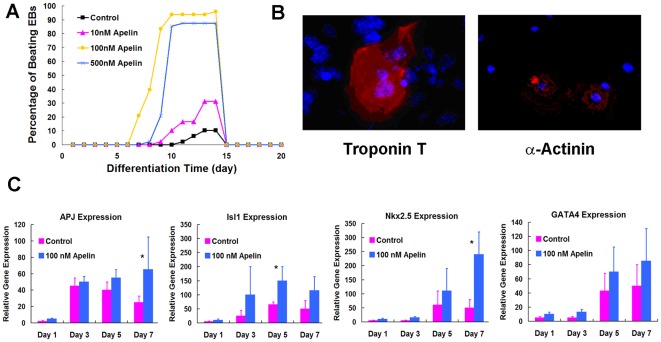
Effect of different concentrations of apelin on mEBs. (A) Percentage of beating EBs, (B) Immunohistochemistry of cardiac troponin T (cTNT, red) and α-actinin on day 14 (red, and blue stain: Hoechst nuclear stain), (C) Quantitative RT-PCR demonstrates significant increase of Isl1 on day 5 and of Nkx2.5 and APJ on day 7 (*p<0.05), and a trend of increase in GATA4 expression.

### Effects of Apelin on mouse and human ESC Cardiac Differentiation

Upon EB formation, the effects of apelin-13 concentration (apelin, 0, 50, 100, 500 nM) on the percentage of contractile EBs, production of cardiac troponin-T (cTNT), connexin 43, and α-actinin (immunohistochemistry, n = 3) and gene expression of cardiac markers (rt-PCR, n = 5) were determined for both mESCs and hESCs up to 28 days. The cell culture medium was changed daily. The percentage of contractile EBs was measured by the number of EBs that showed any degree of contractility divided by the total number of EBs in culture. Chronotropic responses of mEBs to pharmacologic treatment was also examined (n = 5).

**Figure 3:**
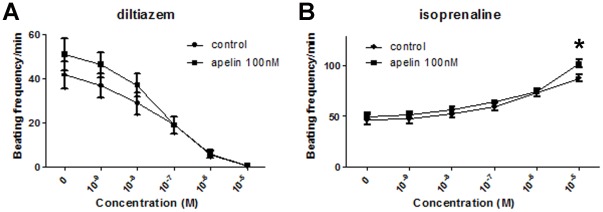
Pharmacologic response of mEBs. (A) Effect of diltiazem (calcium channel blocker) and (B) Isoprenaline (β1-adrenoreceptor agonist) on mESC-derived cardiomyocytes on day 14 of differentiation (*p<0.05). Corresponding negative and positive chronotropy was observed for diltiazem and isoprenaline, respectively.

### Synergistic Effects of Apelin and Mesodermal Differentiation Factors on human ESC Cardiac Differentiation

We adapted and modified two reported cardiac differentiation protocols (Protocols A and B) to examine the synergistic effects of apelin on cardiac differentiation [13], [16]. In Protocol A, the differentiation of hESC-derived EBs (hEBs) was induced with addition of 10 ng/ml BMP-4, 6 ng/ml activin-A and 5 ng/ml bFGF for 5 days, followed by a 4 day treatment period with 10 ng/ml vascular endothelial growth factor (VEGF) and 150 ng/ml dickkopf-related protein 1 (Dkk1). Afterwards, cultures were maintained in 10 ng/ml VEGF and 5 ng/ml Dkk1 to develop mature cardiac phenotype. Basal medium containing 20% FBS was used for the entire culture period in this study. Optimal apelin dosage at 500 nM was administered to Protocol A (Combined Protocol A) from days 0 to 14.

**Figure 4:**
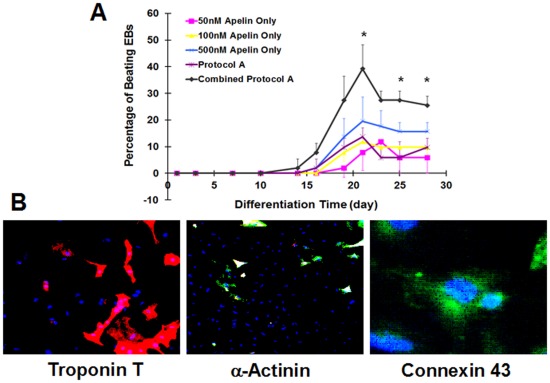
Effects of apelin on hEBs contractility and production of cardiac troponin-T, connexin 43 and α-sarcomeric actin. (A) Combined Protocol A demonstrates highest percentage of contractile hEBs compared to other protocols (*p<0.05 for Combined Protocol A vs. other treatment groups), (B) Immunohistochemistry of cardiac troponin T (red stain), α-actinin (green stain), and connexin-43 (green stain) in 500 nM apelin only group on day 21 (blue stain: Hoechst nuclear stain).

In order to eliminate the unconfined biochemical composition found in the FBS, Protocol B examined cardiac differentiation in a FBS-reduced medium. In Protocol B, single cells of hESCs were aggregated in V-shape well with brief centrifugation in a serum free RPMI1640 basal medium for 2 days with 25 ng/ml BMP-4 and 5 ng/ml bFGF. After the aggregation phase, hEBs were incubated with 20% FBS along with 500 nM of apelin in RPMI1640 basal medium for 2 days. The hEBs were then plated onto matrigel-coated culture surface in a serum free media with 500 nM apelin for 1 additional day. The total exposure of hEBs to apelin was 3 days in Combined Protocol B. The final treatment groups included the following: 1) basal differentiation medium containing 20% FBS, 2) Protocol A, 3) Protocol B, 4) apelin alone, 5) Combined Protocol A, and 6) Combined Protocol B. Percentage of contractile EBs (n = 4), immunohistology of cardiac phenotype (n = 3), and RT-PCR of cardiac-specific genes (n = 5) were assessed serially up to 32 days. Furthermore, in single hESC-derived contractile cardiac cells, live-cell Ca^2+^ imaging was performed on day 21 to measure calcium transients (n = 6) and atomic force microscopy (AFM) was conducted on day 28 to measure the contractile force (n = 4).

**Figure 5:**
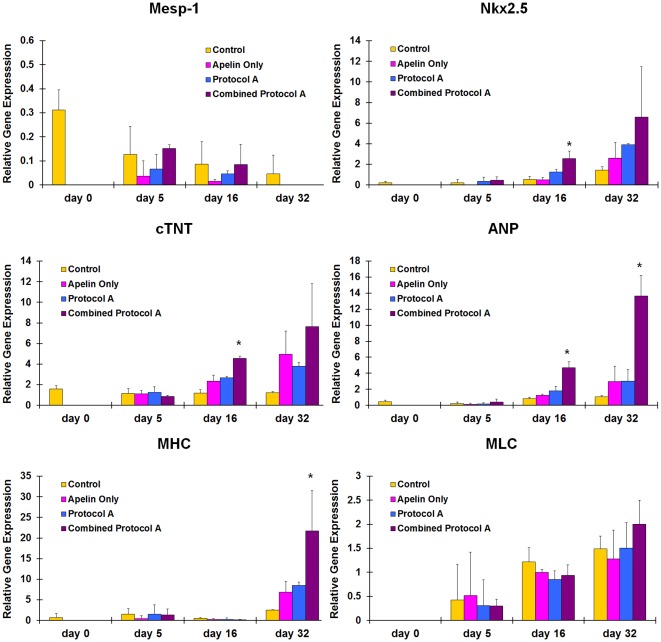
Synergistic effects of apelin on hESC cardiac gene expression using Combined Protocol A. (A) Down-regulation of Mesp-1 is observed in all 4 protocols, (B) Nkx2.5 (an early cardiac transcriptional factor, indicative of cardiac progenitor phenotype) is up-regulated in the combined protocol A at day 16, (C) cTNT (cardiac troponin T, a muscle contractility regulatory protein, indicative of a mature cardiac phenotype) is up-regulated in the combined protocol A at day 16, (D) ANP (atrial natriuretic peptide, a protein peptide secreted by mature cardiomyocyte, indicative of a mature cardiac phenotype) is up-regulated after day 16, (E) MHC (myosin heavy chain, a muscular motor protein, indicative of a mature cardiac phenotype) is up-regulated at day 32, and (F) MLC (myosin light chain, a muscular motor protein, indicative of a mature cardiac phenotype) is up-regulated over time for all groups (*p<0.05 Combined Protocol A vs. other treatment groups).

### Chronotropic Effects of Cardiotonic Drugs

The mEBs were plated onto gelatin coated 48-well plates and allowed to differentiate. One day before the experiment, the cells were fed with 0.5 ml/well differentiation medium and contractile areas were delineated for longitudinal observation. On the day of experiment, the contractile frequency for each area was measured by visual inspection before the addition of the drugs. To examine their pharmacological responses, diltiazem, a calcium channel blocker, and isoprenaline, a β1-adrenoreceptor agonist, were added to the cell culture. The cell cultures were then incubated at 37°C without shaking for 20–30 minutes. The contractile frequency per minute was measured visually. Increasing drug doses (from 0 to 10^−5^ M) were added in the cell cultures followed by 20–30 minutes incubation time. The contractile frequency was then monitored for each drug concentration after the incubation period.

**Figure 6:**
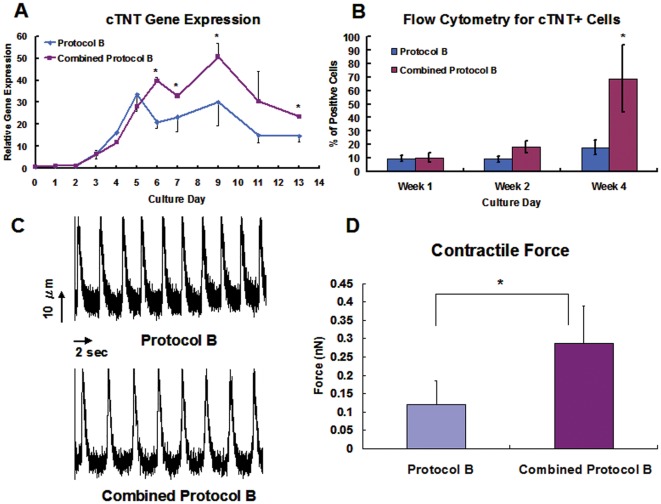
Synergistic effects of apelin on hESC in serum-reduced Combined Protocol B. (A) Significant up-regulation of cTNT gene expression is seen in Combined Protocol B, (B) Significant increase of cTNT^+^ cells is observed in Combined Protocol B, (C) No significant difference in Ca^2+^ transient is seen in the hESC-derived cardiac cells between Combined Protocol B and control groups, and (D) Significant increase in peak contractile force was observed in Combined Protocol B vs. Protocol B (*p<0.05).

### Immunohistochemistry

Cells were fixed with PBS containing 4% paraformaldehyde (Sigma) for 10 minutes at room temperature. After washing with PBS, the cells were permeated using 0.25% Triton X-100 (Sigma) for 10 minutes, then blocked with 10% goat serum (Sigma) for 1 hour at room temperature. Finally, the primary antibodies were incubated for 45 minutes at room temperature. Monoclonal antibodies used in this study included cardiac troponin T (dilution 1∶200, Santa Cruz Biotechnology, Inc., Santa Cruz, CA), Connexin 43 (Sigma), and α-sarcomeric actinin (dilution 1∶500, Santa Cruz Biotechnology). Cells were subsequently counter-stained with Hoechst 33342 (Invitrogen) for nuclear staining. Preparations were examined using fluorescence microscope (Nikon, Japan).

### Gene Expression

Gene expression was measured by reverse transcription followed by real-time polymerase chain reaction (rt-PCR). Primers were designed for mESCs (Nkx2.5, Isl1, and APJ) and hESCs (Mesp-1, Nkx2.5, atrial natriuretic peptide (ANP), cardiac troponin T (cTNT), myosin light chain (MLC) and myosin heavy chain (MHC)). Total RNA was isolated (n = 4) using the Trizol® extraction method (Invitrogen, Carlsbad, CA). The isolated RNA was reverse transcribed into cDNA using the SuperScript™ First-Strand Synthesis System (Invitrogen) and amplified using iQ SYBR Green Supermix (Applied Biosystems) and StepOne Plus Real-Time PCR Detection System (Applied Biosystems). All genes were amplified for 40 cycles. Specific gene expression was first normalized to GAPDH and then compared to the control groups.

### Live-Cell Calcium^2+^ Imaging

Beating EBs from each group were mechanically detached from the tissue culture surface and dissociated with TrypLE Express (Invitrogen) for 5 minutes. Single cells were allowed to attach onto gelatinized glass coverslip (Lab-Tek, Thermo Scientific/Nunc, Rochester, NY) for 5 days prior to the imaging. On the day of imaging, cells were stained with 5 µM Fluo-4 AM and 0.02% Pluronic F-127 (Molecular Probe) for 15 minutes, and fluorescence signal from intracellular Ca^2+^ was monitored using fast line scanning (1.92 ms/line) on a confocal microscope (LSM 510 Meta, Carl Zeiss) with a X63 lens (NA = 1.4) [26].

### Flow Cytometry

At week 1, 2 and 4, hEBs were mechanically detached from the culture surface and dissociated using TryLE Express (Invitrogen) for 5 minutes. Cells were fixed and permeabilized with PharMingen Perm/Fix solution for 30 minutes at 4°C, and incubated with primary antibody against human cTNT (1∶200, Abcam) overnight at 4°C. The next day, cells were stained with fit-C conjugated secondary antibody (1∶500, Abcam) for 30 minutes at room temperature. Expression of markers was determined by FACS Calibur (BD Bioscience) and FlowJo software (Tree Star) to quantify the percentage of cTNT^+^ cells.

### Atomic Force Microscopy

The contractile hEBs were mechanically detached from the tissue culture surface and dissociated with TrypLE Express for 5 minutes. Single hESCs were allowed to attach onto glass bottom plate for 3 days prior to imaging. The culture media of cells were changed to a medium containing 89% Tyrode's buffer, 10% FEB and 1% antibiotic-antimycotic (Cellgro, Mediatech) before the experiment. Cells were maintained at 36°C during the measurement. The contractile force of the cells was measured by AFM (MFP-3D Bio, Asylum Research) using a silicon nitride cantilever (spring constants ∼0.1 N/m, PPP-ContAu, NanoSensors). Cells were gently contacted by the cantilever tip with 400 pN of force. The cantilever tip remained in the position without Z-piezo feedback and the deflection data were collected at a sample rate of 1 kHz for two minutes. The deflection trajectory was converted to force trajectory by multiplying by the spring constant and analyzed by using a Matlab (Mathworks) program. In the force trajectory, the amplitude of beating peaks provided the measurement of cell beating force.

### Statistical Analysis

Data were presented as the mean ± standard deviation. A two-way analysis of variance (ANOVA) was performed to compare the percentage of contractile EBs and gene expression levels between control and experimental groups. The Tukey-Kramer post-hoc test was used for all pair-wise comparisons and statistical significance was set at p<0.05. All statistical analyses were performed using the JMP statistical software package (SAS Institute, Cavy, NC).

## Results

### Effects of Apelin on Mouse and Human ESCs

Apelin concentration was optimized to establish a robust cardiac differentiation protocol. The contractile mEBs were seen as early as day 6 as shown in Figure 2A. The percentage of contractile EBs in all groups peaked around day 14, plateaued around day 16, and decreased around day 18. Compared to control, apelin significantly increased the percentage of contractile EBs in all dosages (p<0.05). Addition of 100 nM of apelin was found to show the highest percentage of beating mESC-derived EBs (mEBs) compared to 50 and 500 nM in concentrations (p<0.05). Immunohistochemistry on single cells derived from 100 nM apelin treated mEBs showed positive production of cardiac troponin-T and α-actinin after 14 days (Fig. 2B). Gene expression analyses revealed up-regulation of Nkx2.5, Isl1 and GATA4, the early cardiac differentiation transcriptional factors, with apelin treatment as early as day 3 (Fig. 2C). The expression of APJ also increased significantly with apelin treatment (Fig. 2C).

The chronotropic effects of the cardiotonic drugs on the *in vitro* function of mEBs between control and apelin treated groups were investigated as shown in Figure 3. Significant negative chronotropic effect on the EBs was observed after the addition of diltiazem, a calcium channel blocker. All contracting EBs ceased to beat at concentration of 10^−5^ M and no difference was found between apelin and control groups (Fig. 3A). When positive chronotropic effect of isoprenaline, a β_1_-adrenoreceptor agonist, was tested, significant increase in the contractile frequency was demonstrated by both control and apelin treated groups. Apelin-treated group showed significantly higher contractile frequency compared to the control group at isoprenaline dose of 10^−5^ M (Fig. 3B).

Similarly, the effects of apelin only on hESC cardiac differentiation were evaluated and the optimal dosage for promoting cardiac differentiation for hESCs was determined. Plated hESC-derived EBs (hEBs) demonstrated contractility as early as day 14 as shown in Figure 4A. The percentage of contractile hEBs in all groups peaked around day 21 and plateaued afterwards. As shown in Figure 4A, a dose dependent difference on the percentage of contractile hEBs was observed in the apelin-only treatment groups. The 500 nM apelin-treated group exhibited the highest percentage of contractile hEBs compared to other concentrations. Consequently, the optimal apelin concentration for establishing a robust cardiac differentiation protocol for hESCs was chosen at 500 nM. This concentration was used to assess the synergistic effects of apelin with Protocols A and B. Positive production of cTNT, connexin-43, and α-sarcomeric actinin by hEBs was found in the apelin treated group after 21 days by immunohistochemistry (Fig. 4B).

### Synergistic Effects of Apelin with BMP-4, Activin-A and bFGF on Human ESCs

In order to establish a robust cardiac differentiation protocol, 500 nM of apelin was combined with other reported mesodermal growth factors for cardiac differentiation (Fig. 1) [16]. When apelin was combined with Protocol A (Combined Protocol A), which includes a long-term exposure of 20% fetal bovine serum (FBS) in the basal culture media, hEBs demonstrated contractility as early as day 14 while other treatment groups started beating after 16–18 days as shown in Figure 4A. Interestingly, after 21 days, hEBs in the Combined Protocol A demonstrated significantly higher percentage of contractile hEBs compared to the basal media control group, apelin only group, and Protocol A (Fig. 4A). Positive production of mature cardiac markers was also found (Fig. 4B). Gene expression of pre-mesodermal marker Mesp-1, early cardiac differentiation marker Nkx2.5, and mature cardiac markers ANP, cTNT, MLC and MHC were quantified over the cell culture period (Fig. 5). The expression of Mesp-1 decreased after 6 days of differentiation in all groups compared to day 0, and was diminished after 32 days except for the basal media differentiation group (Fig. 5). Expression of early cardiac transcriptional factor, Nkx2.5, increased over time for all groups, and was observably up-regulated in the Combined Protocol A compared to all the others after 16 days (Fig. 5). Expression of all mature cardiac markers including ANP, cTNT, MLC and MHC increased over time for all groups. Combined Protocol A significantly increased the gene expression of these mature cardiac markers (Fig. 5).

In order to eliminate the confounding biochemical effects of FBS, the synergistic effects of apelin on cardiac differentiation were further evaluated in combination with serum-reduced Protocol B (Combined Protocol B). It was found that the Combined Protocol B up-regulated the gene expression of cTNT in comparison to Protocol B. This effect of apelin was observed as early as day 5 of the cardiac differentiation (Fig. 6A). The percentage of cTNT^+^ positive cells in culture also increased over time and the addition of apelin significantly increased the cTNT^+^ cells in culture (Fig. 6B). Furthermore, the imaging of Ca^2+^ transients in contractile single cardiac cells in Protocol B vs. Combined Protocol B groups demonstrated characteristic Ca2+ transient and no difference in the contractile frequency or Ca^2+^ transient amplitude between the 2 groups (Fig. 6C). Interestingly, atomic force microscopy data showed significantly higher peak contractile force in the Combined Protocol B compared to Protocol B (Fig. 6D).

## Discussion

We determined the role of apelin-13 (apelin) on cardiac differentiation of mESCs and hESCs and assessed its synergistic effects at both cellular and molecular levels. The optimal dosage of apelin on cardiac differentiation of mESCs and hESCs was examined initially. Then a cardiac differentiation protocol including apelin and mesodermal differentiation factors (BMP-4, activin-A, and bFGF) was established by administering them in a developmentally specific temporal sequence. This study found that apelin promoted cardiac differentiation of both mouse and human ESCs. Furthermore, this novel peptide exerted synergistic effects on the differentiation efficiency when treated with mesodermal differentiation factors and generated a rich homogeneous pool of cardiac cells from the ESCs.

The effects of apelin on the ESCs were initially evaluated through the ability to produce more contractile EBs. The contractile mEBs appeared following 6 days of exposure to apelin, which was significantly earlier than the control group containing 20% FBS. Similarly, an increased percentage of contractile hEBs by apelin was also found. These findings could be attributed to the apelin-induced migration and aggregation of ESCs committed to cardiac lineage, allowing homotypic cellular interactions of mesodermally fated EBs to enhance cardiac differentiation. The 2 populations of cardiac progenitor cells in the development of primary and secondary heart fields are known to migrate and interact to develop into the heart and great vessels [27]–[31]. Myocardial regulatory genes that are activated in both lineages include Gata4-6 (zinc-finger-containing transcription factors of the GATA family), Nkx2.5 (development of first and secondary heart fields), and Isl1 (key transcriptional regulators in the secondary heart field) [32]–[34]. This study demonstrated up-regulation of GATA4, Nkx2.5, and Isl1, indicating enhancement of early cardiac differentiation transcriptional activity. Apelin enhanced the expression of Isl1 on day 3 and both GATA4 and NKX2.5 after day 5 of culture in the mESCs. These findings support the role of apelin in the migration of cardiac progenitor cells into the development of both heart fields. This effect of apelin was confirmed by the higher percentage of cTNT^+^, connexin-43, and α-sarcomeric actin in the apelin-treated EBs, indicating increased homogeneity of the cardiac lineage committed cells.

The study then sought to determine the synergistic effects of apelin in enhancing the cardiac differentiation efficiency of 2 protocols employing mesodermal differentiation factors [13], [16]. Compared to apelin or the two protocols alone, the administration of apelin, BMP-4, activin-A and bFGF significantly enhanced the cardiac differentiation efficiency. Higher percentage of contractile EBs, up-regulation of early and mature cardiac markers, and increased percentage of cTNT^+^ cells confirmed the synergistic effect of apelin in promoting cardiac differentiation. The presence of apelin appears to have promoted the recruitment and aggregation of mesodermally committed progenitor cells [27].

Although induced contractility was not found, a previously published study reported another mechanism involving the up-regulation of myosin light chain 2 L gene expression by apelin [35]. The report suggested that the apelin/APJ signaling was possibly through mitogen-activated protein kinase/p70S6 pathway downstream of Cripto [35]. In other studies, it was demonstrated that apelin did not modulate L-type Ca^2+^ or voltage-activated K^+^ currents in isolated adult rat ventricular myocytes and did not change the intracellular baseline calcium level [36], [37]. These data are consistent with this study's findings regarding the Ca^2+^ transient data in which the exposure of apelin on hESCs significantly increased the peak contractile force while the Ca^2+^ transient remained unchanged. Other studies found that apelin-13 was the predominant isoform in cardiac tissue and the most potent endogenous inotropic agents tested [38], [39]. Addition of apelin was shown to increase the sarcomere shortening by 1.4 fold in single mature cardiomyocytes resulting in increased contractility of a whole rat heart and the atrial strips [40]. These results support our finding that apelin not only enhanced the differentiation and maturation of ESC-derived cardiac cells, but also enhanced their contractile function. Although the mechanism of inotropic actions of apelin remains unclear, it has been found to be independent of angiotensin II, endothelin-1, catecholamines and nitric oxide release [37]. Some studies suggested that it might be related to the increased availability of activator Ca^2+^ and/or increased Ca^2+^ responsiveness [38], [39]. Although some studies reported a marginal increase in calcium transient in mature cardiomyocytes with the addition of apelin in vitro, many studies have found no effect of apelin on Ca^2+^ availability [36], [37], [39], [41]. Finally, It was reported that the effect of apelin on rat myocyte contractility was modulated through the sacrolemmal sodium-hydrogen ion exchanger which led to sensitization of myofilaments to Ca^2+^ [42]. These findings are consistent with our data that the Ca^2+^ transient did not change between the apelin-treated and non-treated groups.

In conclusion, this study demonstrated that apelin enhanced cardiac differentiation when combined with the mesodermal differentiation factors, BMP-4, activin-A and bFGF. The underlying mechanism could be attributed to the recruitment and enhanced aggregation of the mesodermal progenitor cells. The Combined Protocols A and B systematically generated a more homogeneous population of cardiac lineage committed cells. Isolation of the cells at an earlier time point may possibly yield a pool of cardiac lineage committed progenitor cells. Future studies will establish an effective cell sorting strategy to select the cardiac progenitor cells.
